# Quantification of intrusive/retraction force and moment generated during en-masse retraction of maxillary anterior teeth using mini-implants: A conceptual approach

**DOI:** 10.1590/2177-6709.22.5.047-055.oar

**Published:** 2017

**Authors:** A. Sumathi Felicita

**Affiliations:** 1 Saveetha University, Saveetha Dental College, Department of Orthodontics and Dentofacial Orthopedics (Chennai, India).

**Keywords:** Orthodontics, Orthodontic space closure, Orthodontic anchorage procedures

## Abstract

**Objective:**

The aim of the present study was to clarify the biomechanics of en-masse retraction of the upper anterior teeth and attempt to quantify the different forces and moments generated using mini-implants and to calculate the amount of applied force optimal for en-masse intrusion and retraction using mini-implants.

**Methods:**

The optimum force required for en-masse intrusion and retraction can be calculated by using simple mathematical formulae. Depending on the position of the mini-implant and the relationship of the attachment to the center of resistance of the anterior segment, different clinical outcomes are encountered. Using certain mathematical formulae, accurate measurements of the magnitude of force and moment generated on the teeth can be calculated for each clinical outcome.

**Results:**

Optimum force for en-masse intrusion and retraction of maxillary anterior teeth is 212 grams per side. Force applied at an angle of 5^o^ to 16^o^ from the occlusal plane produce intrusive and retraction force components that are within the physiologic limit.

**Conclusion:**

Different clinical outcomes are encountered depending on the position of the mini-implant and the length of the attachment. It is possible to calculate the forces and moments generated for any given magnitude of applied force. The orthodontist can apply the basic biomechanical principles mentioned in this study to calculate the forces and moments for different hypothetical clinical scenarios.

## RESUMO

**Objetivo:**

o objetivo do presente estudo foi explicar o funcionamento da biomecânica de retração em massa dos dentes anterossuperiores e tentar quantificar as diferentes forças e momentos gerados ao se usar mini-implantes, além de calcular a quantidade ideal de força a ser aplicada para se obter a intrusão/retração em massa ancorada em mini-implantes.

**Métodos:**

a força ideal necessária para se realizar a intrusão/retração em massa pode ser calculada por meio de fórmulas matemáticas simples. Dependendo da posição do mini-implante e da posição do acessório, a ele conectado, com relação ao centro de resistência do segmento anterior, obtêm-se desfechos clínicos diferentes. Aplicando certas fórmulas matemáticas, pode-se calcular com precisão a magnitude da força e do momento gerado nos dentes, para cada desfecho clínico.

**Resultados:**

a força ideal para se obter a intrusão/retração em massa dos dentes anterossuperiores é de 212 gramas por lado. A força aplicada em um ângulo de 5° a 16° em relação ao plano oclusal produz componentes de força de intrusão/retração que se encontram dentro dos limites fisiológicos.

**Conclusão:**

podem ser encontrados diferentes desfechos clínicos dependendo da posição do mini-implante e do comprimento do acessório. É possível calcular as forças e os momentos gerados para qualquer magnitude de força aplicada. Assim, o ortodontista clínico pode aplicar os princípios biomecânicos básicos apresentados nesse estudo para calcular as forças e os momentos em diferentes cenários clínicos hipotéticos.

## INTRODUCTION

**Figure 1:**
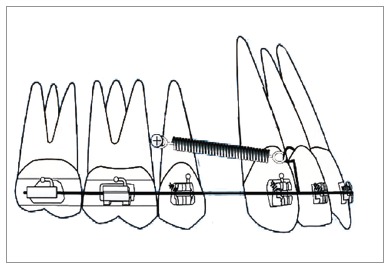
Force applied from the mini-implant placed between the maxillary second premolar and first molar to an attachment soldered onto the archwire distal to the lateral incisor.

**Figure 2:**
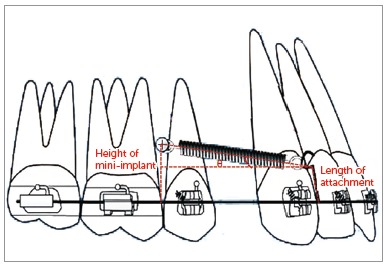
Showing the angulation made by the applied force to the horizontal, depending on the height of the soldered attachment and the height of the mini-implant from the base archwire.

Space closure with mini-implant anchorage usually involves application of force by means of closed coil spring or elastic traction from the mini-implant placed between the maxillary second premolar and maxillary first molar, bilaterally^1^ ^-^ ^7^, to an attachment placed between the lateral incisor and canine, on a continuous archwire (Fig 1). This usually results in the application of a diagonal vector of force on the maxillary anterior teeth of both sides. This applied diagonal force vector can be resolved into an intrusive and retraction component, and its magnitude depends on the direction of applied force, in relation to the occlusal plane. This direction of applied force is determined by the length of the attachment and the height of the mini-implant from the base archwire (Fig 2). Therefore, the direction of applied force becomes more obtuse as the length of the attachment is reduced and the height of the mini-implant is increased, and vice versa. 

However, retraction on a continuous archwire produces a statically indeterminate system and cannot be quantified. But if the base archwire is segmented distal to the canine on both sides, it is possible to produce a statically determinate force system that can be accurately calculated. This paper deals with a statically determinate force system. Thus, the aim of this paper is:

1. To calculate the magnitude of optimum force required for en-masse retraction using mini-implants.
2. To determine the optimum direction of applied force to produce optimum resultant forces.
3. To quantify the magnitude of force and moment generated for any given clinical outcome.

## MATERIAL AND METHODS

**Figure 3:**
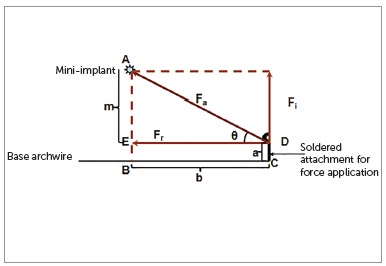
Showing the parallelogram law of vectors with f_a_ being the resultant force of the two individual forces f_i_ and f_r_.

**Figure 4:**
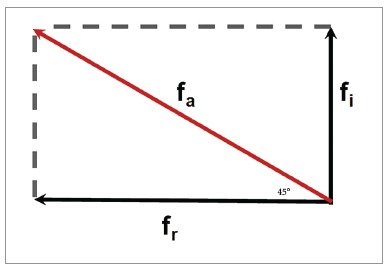
Showing f_a_ as the hypotenuse of a right angled triangle with the component vectors f_i_ and f_r_ forming the other two sides of the triangle.

The direction of force application from the maxillary anterior teeth to the mini-implant can be represented using a simple mathematical model (Fig 3). This model is designed based on the parallelogram law of forces. According to the parallelogram law of vectors, a parallelogram has two adjacent sides, that represent two force vectors; and a diagonal, which is the resultant sum of the two vectors. For all practical purposes, resolution of a diagonal force in orthodontics is done with two vectors perpendicular to each other,^9^ and the direction of resultant force at an angle of 45° to the occlusal plane (Fig 4).

According to this model, the magnitude of applied force can be calculated using the formula:^9^ F_a_ = √ (f_i_ ^2^ + f_r_ ^2^). 

The optimum force for simultaneous intrusion and retraction of anterior teeth = √ (210^2^+ 30^2^) =√ (44,100 + 900) = 212.132 g, since optimum force required to translate a single anterior tooth is 70g^8^ and that required to intrude one anterior teeth is 10g^8^.

However, in a clinical situation the force is not always applied at 45° to the occlusal plane and varies depending on the length of attachment and height of the mini-implant from the occlusal plane. Hence for any given clinical situation the direction of applied force to the occlusal plane can make an angle ‘θ’ which can vary from 0° to 90°. This angle can be derived either by the direct method or indirect method.

### Direct method

**Figure 5:**
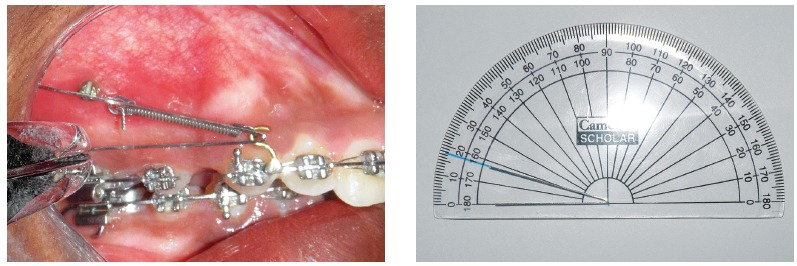
Direct measurement of the angle made by the applied force to the occlusal plane.

Bend a stiff straight length 0.07” round wire. The bent wire is adjusted such that the upper end of the bent wire is placed on the coil spring, the vertex of the bent wire is placed on the point of force application on the attachment and the lower end of the bent wire is placed parallel to the base archwire. This angulation obtained is measured to the nearest degree (Fig 5). 

### Indirect method

**Figure 6:**
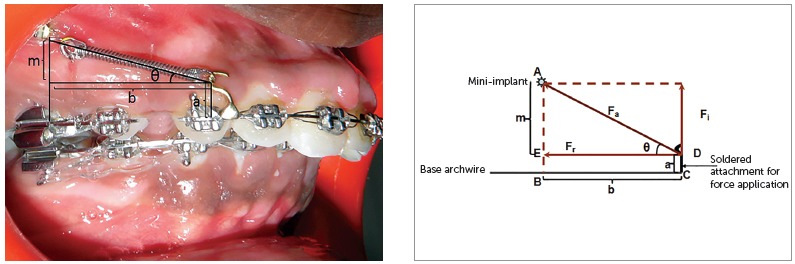
Indirect measurement of the angle made by the applied force to the occlusal plane.

The angulation of the applied force can also be obtained from certain intraoral linear measurements. The following measurements are made, as shown in Figures 6A and 6B: AE, denoted as ‘m’; EB = DC, denoted as ‘a’; and BC = ED, denoted as ‘b’ - where ‘m’ is the perpendicular distance in millimeters from the mini-implant to the base archwire; ‘a’ is the length of the attachment in millimeters from the point of attachment on the base archwire to the point of attachment of the coil spring; and ‘b’ is the linear horizontal distance between the perpendicular from the mini-implant to the base archwire and the attachment on the base archwire.

Now, according to Figure 6:

» AE= AB minus EB=AB minus DC (since EB=DC);
» AED is a right angled triangle with an angle θ between AD and ED. To determine ‘θ’:
» θ = Cos (adjacent side/hypotenuse) = Sin (opposite side/ hypotenuse) = tan (opposite side/ adjacent side).

Using any of the aforementioned equations the value of ‘θ’ can be determined: ‘θ’ gives the direction of applied force.

However, the intrusive and retraction forces generated for any given direction ‘θ’ of the applied force may not be optimum, as shown in Table 1, and it is imperative to determine the optimum direction of applied force from the mini-implant to the anterior teeth. 

**Table 1:** The intrusive and retraction force generated for a diagonal force of 212 grams for different angulations.

Degrees	Intrusive force (grams)	Retraction force (grams)
0	0	212
5	18.4864	211.1944
6	22.154	210.834
7	25.8428	210.41
8	29.5104	209.9436
9	33.1568	209.3924
10	36.8032	208.7776
11	40.4496	208.0992
12	44.0748	207.3572
13	47.7	206.5728
14	51.2828	205.7036
15	54.8656	204.7708
16	58.4272	203.7956
17	61.9888	202.7356
18	65.508	201.6332
19	69.0272	200.446
20	72.504	199.2164
30	106	183.592
40	136.2736	162.392
50	162.392	136.2736
60	183.592	106
70	199.2164	72.504
80	208.7776	36.8032
90	212	0

The value of 212 g is the optimum force applied from the attachment on the base archwire to the mini-implant. The magnitude of applied force can be accurately measured using a Dontrix gauge (TP Orthodontics, Inc.) or a Corex gauge (Haag- Streit AG, Gartenstadstrasse 10, 3098 Koeniz, Switzerland).

Theoretically, the direction of this force can range from 0° to 90°. Using the same mathematical model as above, the resultant forces for a clinical situation as shown in Figure 3 can be calculated. For a given clinical situation where the optimum diagonal force of 212 g per side is at 16° to the horizontal - as determined using either the direct or the indirect method -, the retraction and intrusive force generated can be calculated as follows:

F_r_ = F x Cos θ 

Retraction force = F_r_ = 212 x Cos 16 

 = 212 x 0.2756

 = 58.4272 g

Therefore, F_i_ = F x Sin θ 

F_i_ = Intrusive force = 212 x Sin 16 

 = 212 x 0.9613 

 = 203.7956 g 

Table 1 gives the intrusive and retraction force generated for an optimum force of 212 g for different angulations. It can be noted that the intrusive component of force is beyond the physiologic limit for an angulation of 17° with an intrusive force of 61.98888 g.

The optimum force and the optimum direction of force application have now been established. But in a clinical situation the applied force does not always pass through the center of resistance of the maxillary anterior teeth and moments are generated depending on the relation between the direction of applied force and the center of resistance of the anterior teeth. The magnitude of these moments is given as:

» Moment of a given force = Distance between the CRes and the point of force application x Force.
» The moment generated by the horizontal component of force = Vertical Distance from the CRes to the point of force application x horizontal force (calculated retraction force at an angle of 16°)

**Figure 7:**
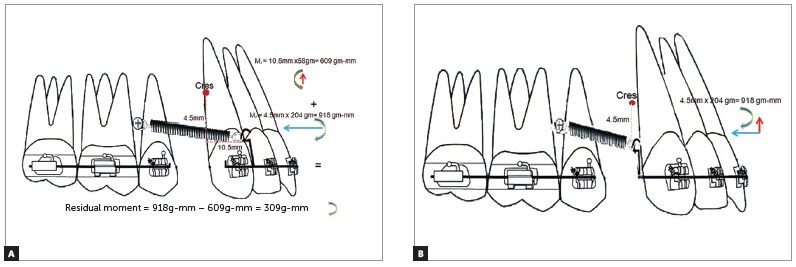
A) Calculation of residual moment when the attachment is placed between the lateral incisor and canine. B) Calculation of moment when the attachment is placed distal to the canine.

= M_r_ = 4.5 mm x 204 g = 918 g-mm per side (Fig 7A). The moment generated by the vertical component of force = Horizontal Distance from the CRes to the point of force application x vertical force (calculated intrusive force at 16°)

= M_i_ = 10.5 mm x 58 g = 609 g-mm per side (Fig 7A). 

The vertical and horizontal distance was determined from the vertical and sagittal position of the CRes. The center of resistance varies from 8.1 mm to 14.7 mm from the incisal edge in the vertical direction when a sagittal force is applied on the six anterior teeth.^10^ The length of the attachment determines the distance between the CRes and the point of force application. In the described situation the attachment was placed 4.5 mm incisal to the CRes. 

In the sagittal direction the center of resistance is located on a line 3 mm behind the distal surface of the maxillary canine, when a vertical force is applied to the six anterior teeth^11^. Since the mesiodistal width of the upper permanent canine in Caucasians is 7.5-8 mm^12^ the center of resistance lies 10.5-11mm distal to the attachment, in the sagittal direction. 

Since the retraction force is occlusal to the center of resistance, a clockwise moment is generated by the retraction force and the intrusive force is labial to the CRes, an anticlockwise moment is generated.

Therefore:

» residual moment = difference between clockwise and anticlockwise moments =
» 918 g-mm - 609 g-mm = 309 g-mm per side.

Thus, the total residual moment produced is a clockwise moment of 309 g-mm per side at an angle of 16°. Tipping of the maxillary anterior teeth may be expected due to the clockwise moment. The moment generated for varying lengths of attachment can also be calculated. 

If the point of force application is placed distal to the canine, 4.5 mm occlusal to the CRes, the intrusive force passes through the CRes and a clockwise moment of 918 g-mm is created because of the retraction force (Fig 7B).

## RESULTS

The amount of 212 grams is the optimum force required for en-masse intrusion and retraction of anterior teeth using mini-implants. 

However, the resultant forces are optimum when the direction of force application ranges from 5° to 16°.

A residual moment of 309 g was produced when an optimum force of 212 g was applied at 16° to the occlusal plane when the attachment was placed between the lateral incisor and canine.

A residual clockwise moment of 918 g was produced when an optimum force of 212 g was applied at 16° to the occlusal plane when the attachment was placed distal to the canine.

The residual moment generated is definitely smaller when the attachment is placed between the lateral incisor and canine, as compared to that placed distal to the canine.

## DISCUSSION

The different clinical outcomes encountered during en-masse retraction using mini-implants can be classified into three types, depending on the relation between the point of force application and the center of resistance of the maxillary anterior teeth. 

» Outcome I: The point of force application lies apical to the center of resistance of the maxillary anterior teeth (There are two sub-types, depending on the relation between the mini-implant and the point of force application).

**Figure 8:**
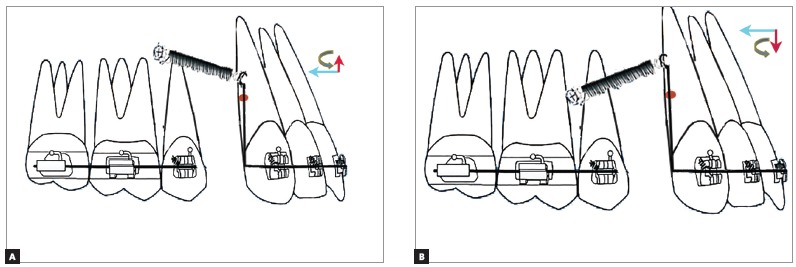
A) Outcome IA: The point of force application is located apical to the CRes of the maxillary anterior teeth but occlusal to the mini-implant. B) Outcome IB: The point of force application is located apical to the CRes of the maxillary anterior teeth and also apical to the mini-implant.

» Outcome IA: When the point of force application is located apical to the center of resistance of the maxillary anterior teeth and occlusal to the mini-implant, a counter-clockwise moment is generated along with an intrusive and retraction component of force (Fig 8A). This counter-clockwise moment will result in labial flaring of the teeth, with bite-opening augmenting the effect of the mild intrusive force component. The magnitude of retraction force component should be sufficient to overcome the flaring of the anterior teeth prior to retraction. 

» Outcome IB: When the point of force application is apical to the center of resistance and the mini-implant, a large counter-clockwise moment is generated, with an extrusive and retraction component of force (Fig 8B). The large counter-clockwise moment can cause severe labial flaring with associated bite opening, which may be partly negated by the mild extrusion caused by the extrusive component of force. Hence only retraction of teeth can be expected. The retraction force has to overcome the labial flaring of the teeth to allow them to be retracted. 

» Outcome II: The point of force application lies on the CRes of the maxillary anterior teeth; if the point of force application lies on the center of resistance of the maxillary anterior teeth, bodily movement of maxillary anterior teeth occurs (there are the following three situations, depending on the position of the mini-implant and the attachment).

**Figure 9:**
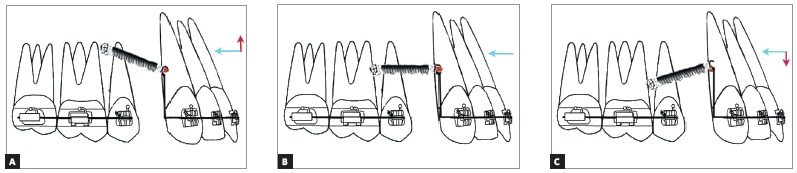
A) Outcome IIA: The point of force application lies on the CRes of the maxillary anterior teeth occlusal to the mini-implant. B) Outcome IIB: The point of force application lies on the CRes of the maxillary anterior teeth at the level of the mini-implant. C) Outcome IIC: The point of force application lies on the CRes of the maxillary anterior teeth apical to the mini-implant.

» Outcome IIA: If the mini-implant is apical to the center of resistance, an intrusive and retraction force is generated, without any moment (Fig 9A). True intrusion and translation can be expected.

» Outcome IIB: If the mini-implant is at the level of center of resistance, only a retraction force is generated, without any moment (Fig 9B). If the implant is in the same plane of the center of resistance and the force passes through the center of resistance, only translation with no intrusion will take place.

» Outcome IIC: If the mini-implant is occlusal to the center of resistance, an extrusive and retraction force is generated, without any moment (Fig 9C). True extrusion and translation will occur.

» Outcome III: The point of force application lies occlusal to the CRes of the maxillary anterior teeth (there are the following three types, depending on the relation between the mini-implant and the point of force application).

**Figure 10:**
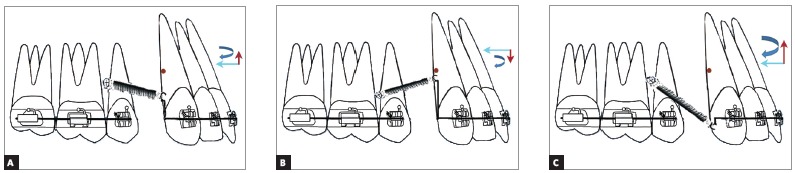
A) Outcome IIIA: The point of force application lies occlusal to the mini-implant and the CRes of the maxillary anterior teeth. B) Outcome IIIB: The point of force application lies apical to the mini-implant occlusal to the CRes of the maxillary anterior teeth. C) Outcome IIIC: The point of force application lies occlusal to the mini-implant but away from the CRes of maxillary anterior teeth slightly occlusal to the base archwire.

» Outcome IIIA: If the point of force application is occlusal to the mini-implant and the center of resistance of the maxillary anterior teeth and apical to the base archwire, an intrusive and retraction force is generated along with a clockwise moment (Fig 10A). This will result in lingual tipping of the teeth, with bite deepening. However, this may be negated by the intrusive force component. Therefore, only retraction of the teeth will be attained. 

Outcome IIIB: If the point of force application lies apical to the mini-implant and occlusal to the center of resistance of the maxillary anterior teeth, but apical to the occlusal plane, an extrusive and retraction force along with a clockwise moment is generated (Fig 10B). This extrusive force along with a clockwise moment can cause bite deepening in the anterior region during retraction. 

» Outcome IIIC: If the point of force application is at the level of the occlusal plane and occlusal to the center of resistance of the maxillary anterior teeth, a large clockwise moment is generated. A greater intrusive force is generated along with a retraction force (Fig 10C).

Since the point of force application is far away from the CRes of the maxillary anterior teeth, a greater clockwise moment is generated. However, the intrusive force may not be sufficient to completely negate the bite deepening tendency that may occur due to the clockwise moment, and bite deepening may occur. Application of force closer to the center of resistance of the maxillary anterior teeth will reduce the moment. This is often beneficial. Hence, Outcome IIIC may not be appropriate from a biomechanical stand point. 

Thus, it can be inferred that if a greater intrusive force component is desired, the force should be applied away from the occlusal plane, and vice versa. If a greater retraction force component is required, applied force should be placed closer to the occlusal plane, and vice versa. It can be stated that pure translation and true intrusion or extrusion of the teeth can be attained only for the configuration in Outcome II. In Outcomes I and III, tipping is expected to occur depending on the magnitude of moment generated. The clinician can adjust the force magnitude according to the intended type of orthodontic movement. 

A number of studies^13^ ^-^ ^18^ have been done on the center of resistance of the maxillary anterior teeth, and a large variability in the position of the center of resistance was recorded over time, even for the same tooth. As a result, close monitoring of the dental movement is required.

This study in its entirety is a theoretical one and is based on well-known mathematical and physical formulae. Application of these situations in clinical practice depends on a number of factors like variability in the position of center of resistance, bone heights, root lengths, patient biology, root surface area, binding friction, etc. It would be much more relevant to the practicing orthodontist to apply the basic biomechanical principles mentioned in this study to calculate the forces/moments prior to the commencement of treatment.

The length and position of the attachment is important in determining the magnitude of moment generated. The length of attachment can be limited by the depth of the vestibule, as a relatively long attachment can cause soft tissue irritation and ulceration. It is better to place the attachment between the lateral incisor and canine as lesser residual forces are produced, in comparison to that placed distal to the canine.

It is to be noted that all the mechanics discussed above are for statically determinate system. If the same principles are applied during en-masse retraction on a continuous archwire, a change in the inclination of the occlusal plane can occur because of the moments produced. When retraction using mini-implants in done on a continuous archwire, it may not be possible to accurately predict the magnitude of force generated and its effect on the dentition.

## CONCLUSION

1. Optimum force for en-masse intrusion and retraction using mini-implants is 212g per side.
2. Forces applied at an angle of 5° to 16° to the occlusal plane produces force components within the physiologic limit.
3. An attachment placed between the lateral incisor and the canine result in lesser residual moments and is therefore a better biomechanically efficient system.
4. Different clinical outcomes will result depending on the height of the mini-implant and the length of the attachment, which will generate an intrusive/extrusive and retraction component of force along with a clockwise or counter-clockwise moment, depending on its relation to the center of resistance of the anterior teeth.

## References

[1] Park HS, Jeong SH, Kwon OW (2006). Factors affecting the clinical success of screw implants used as orthodontic anchorage. Am J Orthod Dentofacial Orthop.

[2] Kyung HM, Park HS, Bae SM, Sung JH, Kim IB (2003). Development of orthodontic micro-implants for intraoral anchorage. J Clin Orthod.

[3] Moon CH, Lee DG, Lee HS, Im JS, Baek SH (2008). Factors associated with the success rate of orthodontic miniscrews placed in the upper and lower posterior buccal region. Angle Orthod.

[4] Baumgaertel S, Razavi MR, Hans MG (2008). Mini-implant anchorage for the orthodontic practitioner. Am J Orthod Dentofacial Orthop.

[5] Herman RJ, Currier GF, Miyake A (2006). Mini-implant anchorage for maxillary canine retraction a pilot study. Am J Orthod Dentofacial Orthop.

[6] Chung KR, Nelson G, Kim SH, Kook YA (2007). Severe bidentoalveolar protrusion treated with orthodontic microimplant-dependent en-masse retraction. Am J Orthod Dentofacial Orthop.

[7] Garfinkle JS, Cunningham LL, Beeman CS, Kluemper GT, Hicks EP, Kim MO (2008). Evaluation of orthodontic mini-implant anchorage in premolar extraction therapy in adolescents. Am J Orthod Dentofacial Orthop.

[8] Proffit WR, Fields HW (2000). Contemporary orthodontics.

[9] Gross D, Hauger W, Schröder J, Wall W, Rajapakse N, Schroder JR (2013). Engineering Mechanics 1: Statics.

[10] Reimann S, Keilig L, Jäger A, Bourauel C (2007). Biomechanical finite-element investigation of the position of the centre of resistance of the upper incisors. Eur J Orthod.

[11] Pedersen E, Isidor F, Gjessing P, Andersen K (1991). Location of centres of resistance for maxillary anterior teeth measured on human autopsy material. Eur J Orthod.

[12] Ash N (2003). Wheeler's Dental anatomy, physiology and occlusion.

[13] Vanden Bulcke MM, Dermaut LR, Sachdeva RC, Burstone CJ (1986). The center of resistance of anterior teeth during intrusion using the laser reflection technique and holographic interferometry. Am J Orthod Dentofacial Orthop.

[14] Vanden Bulcke MM, Burstone CJ, Sachdeva RC, Dermaut LR (1987). Location of the centers of resistance for anterior teeth during retraction using the laser reflection technique. Am J Orthod Dentofacial Orthop.

[15] Choy K, Kim KH, Burstone CJ (2006). Initial changes of centres of rotation of the anterior segment in response to horizontal forces. Eur J Orthod.

[16] Jeong GM, Sung SJ, Lee KJ, Chun YS, Mo SS (2009). Finite element investigation of the center of resistance of the maxillary dentition. Korean J Orthod.

[17] Lee HK, Chung KR (2001). The vertical location of the center of resistance for maxillary six anterior teeth during retraction using three dimensional finite element analysis. Korean J Orthod.

[18] Sia S, Koga Y, Yoshida N (2007). Determining the center of resistance of maxillary anterior teeth subjected to retraction forces in sliding mechanics an in vivo study. Angle Orthod.

